# Action Video Gaming Does Not Influence Short-Term Ocular Dominance Plasticity in Visually Normal Adults

**DOI:** 10.1523/ENEURO.0006-20.2020

**Published:** 2020-05-21

**Authors:** Xiaoxin Chen, Shijia Chen, Deying Kong, Junhan Wei, Yu Mao, Wenman Lin, Yiya Chen, Zhimo Yao, Seung Hyun Min, Fan Lu, Jia Qu, Robert F. Hess, Jiawei Zhou

**Affiliations:** 1 State Key Laboratory of Ophthalmology, Optometry and Vision Science, School of Ophthalmology and Optometry and Eye Hospital, Wenzhou Medical University, Wenzhou 325027, China; 2 McGill Vision Research, Department of Ophthalmology and Visual Sciences, McGill University, Montreal, Quebec H3G 1A4, Canada

**Keywords:** action video gaming, binocular phase combination, monocular patching, ocular dominance, visual plasticity

## Abstract

Action video gaming can promote neural plasticity. Short-term monocular patching drives neural plasticity in the visual system of human adults. For instance, short-term monocular patching of 0.5–5 h briefly enhances the patched eye’s contribution in binocular vision (i.e., short-term ocular dominance plasticity). In this study, we investigate whether action video gaming can influence this plasticity in adults with normal vision. We measured participants’ eye dominance using a binocular phase combination task before and after 2.5 h of monocular patching. Participants were asked to play action video games, watch action video game movies, or play non-action video games during the period of monocular patching. We found that participants’ change of ocular dominance after monocular patching was not significantly different either for playing action video games versus watching action video game movies (Comparison 1) or for playing action video games versus playing non-action video games (Comparison 2). These results suggest that action video gaming does not either boost or eliminate short-term ocular dominance plasticity, and that the neural site for this type of plasticity might be in the early visual pathway.

## Significance Statement

Recent studies have shown that short-term (0.5–5 h) monocular patching induces a new form of short-term ocular dominance plasticity in human adults, in which the patched eye rather than the unpatched eye gets stronger, and the effect is transient. On the other hand, there is evidence that action video gaming has potential in enhancing perceptual learning induced visual plasticity in adulthood. In this study, we found that action video gaming did not impact short-term ocular dominance plasticity in visually normal adults. Our psychophysical evidence suggests that the neural site of this plasticity should be local and early in the cortical pathway.

## Introduction

Action video gaming has been popular in the general public and for research (Green and Bavelier, 2003, 2006, 2007, 2012; Dye et al., 2009; Buckley et al., 2010; Jeon et al., 2012; Appelbaum et al., 2013; Latham et al., 2013; Morin-Moncet et al., 2016; Franceschini et al., 2017; Bediou et al., 2018; Föcker et al., 2018). It is fast-paced and perceptually demanding, requiring the players to provide quick motor responses and oversee objects in surroundings (Dale and Green, 2017; Bediou et al., 2018; Wong and Chang, 2018; Bavelier and Green, 2019). In both observational (Green and Bavelier, 2003; Blacker and Curby, 2013; Wilms et al., 2013; Huang et al., 2017) and training (Green and Bavelier, 2003; Boot et al., 2008; Oei and Patterson, 2013; Bisoglio et al., 2014) studies, it has been shown to enhance our cognition, perception and attention on task-relevant and irrelevant visual stimuli (Green and Bavelier, 2003, 2006; Castel et al., 2005; Boot et al., 2008; Dye et al., 2009; Wang et al., 2016; Föcker et al., 2018; Bavelier and Green, 2019). Action video gaming also improves visual functions of adults. For instance, after 30–50 h of action video game training, the adults exhibited enhanced spatial resolution (Green and Bavelier, 2007), improved contrast sensitivity (Li et al., 2009), and better performance in a visual counting task (Li et al., 2011). Jeon et al. (2012) later confirmed these visual improvements by measuring visual acuity, stereopsis, global motion, and configural face processing. Electrophysiological evidence shows that professional gamers have faster detection and responses to visual stimuli (Latham et al., 2013). Taken together, these studies suggest that action video gaming has potential in enhancing visual plasticity in adulthood.

Recently, a new form of neural plasticity has been reported in adults. Patching one eye (i.e., monocular patching) for a short period (0.5–5 h) of time increases the contribution of the patched eye in binocular vision (Lunghi et al., 2011; Zhou et al., 2013a,b; Bai et al., 2017; Kim et al., 2017; Min et al., 2018, 2019; Ramamurthy and Blaser, 2018). The change, which is referred to as short-term ocular dominance plasticity (Lunghi et al., 2015a), is linked to the primary visual cortex (Zhou et al., 2017). The plasticity is quite different from that observed during the critical period, where the unpatched eye improves (Hubel and Wiesel, 1970; Berardi et al., 2000). Lunghi et al. (2011) first reported the phenomenon using binocular rivalry (i.e., binocular competition); the change lasted for up to 90 min. Other investigators subsequently confirmed this finding via binocular rivalry or binocular combination (Zhou et al., 2013a,b; Bai et al., 2017; Kim et al., 2017; Başgöze et al., 2018; Ramamurthy and Blaser, 2018). The neural basis of this short-term ocular dominance plasticity is thought to occur in the early visual pathway (Lunghi et al., 2015a,b; Zhou et al., 2015; Chadnova et al., 2017; Binda et al., 2018). Despite these numerous studies, the neural mechanisms of this plasticity and factors that could enhance it are still unknown.

Recent studies (Lunghi et al., 2016; Sauvan et al., 2019) suggest that neuroplastic changes induced by both short-term and long-term patching are tightly connected. With a presumably similar neural mechanism, one form of plasticity might be able to predict or enhance the other. Given the potential of action video gaming in enhancing the long-term visual plasticity as we mentioned above (e.g., perceptual learning; Li et al., 2009, 2011; Jeon et al., 2012), we thought it would be worthwhile to see whether action video gaming could influence short-term ocular dominance plasticity. In particular, we asked our participants to complete three different tasks (playing action video games, watching action video game movies and playing non-action video games) during 2.5 h of monocular patching, and compared their pre-patching and post-patching ocular dominance. We hypothesized that action video gaming could strengthen this form of plasticity, and, therefore, we expected ocular dominance to be modulated significantly more with action video gaming, compared with the other two tasks. We found that for all three conditions, monocular patching induced significant changes in ocular dominance. However, our results showed no evidence for a strengthened effect with action video gaming.

## Materials and Methods

### Participants

We recruited twelve normal adults (age: 23.00 ± 2.05 years old; seven females) for this study. According to their reported playing habits and the ranks given by their games, four of the participants were considered “expert” whereas the other eight were considered “less experienced”. We did not recruit “novices” (with no experience of the action video games that we used) in this study. This was because the gaming tasks could be difficult for novices to finish due to the complexity of this game genre (e.g., the complicated environment and the manipulation of items and/or spells in these games). All participants had normal or corrected-to-normal visual acuity (no worse than 0.0 logMAR) in both eyes, with spherical equivalent no more than 1.00D and astigmatism no more than 0.75D. No participants had ophthalmic diseases including but not limited to strabismus, amblyopia and nystagmus, or had history of visual training, occlusion therapy or ophthalmic surgeries including recently-performed refractive surgeries. During the experiments, participants were required to wear their normal refractive correction if needed.

A written informed consent was obtained from each participant before the beginning of the experiments. This study was in line with the Declaration of Helsinki and was approved by the Ethics Committee of Wenzhou Medical University.

### Apparatus

In the ocular dominance measures, all stimuli were generated on a MacBook Pro (13-inch, 2017, Apple Inc) running MATLAB R2016b (The MathWorks Inc) with Psychtoolbox 3.0.14 extensions. We used a head-mounted display, GOOVIS (AMOLED display, NED Optics), to achieve dichoptic viewing. The refresh rate of the display was 60 Hz, and the resolution was 1920 × 1080. Gamma correction was applied to ensure a linear output in the test. We used a custom-made chinrest to prevent movements of participants’ heads during the measurement sessions.

We included two desktop games, the League of Legends (Riot Games) and the PlayerUnknown’s Battlegrounds (PUBG Corporation), and their similar mobile versions (Tencent Games; for details, see https://pvp.qq.com, https://pubgm.qq.com, and https://pg.qq.com) as action video games in this study. For non-action video games, we included Minesweeper (http://minesweeperonline.com/). Participants used their own devices (either laptops or mobile devices) for all tasks.

### Design

**Figure 1.**
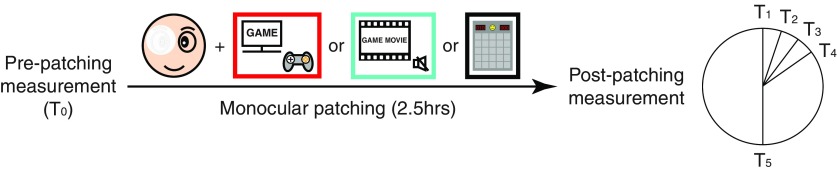
An illustration of the experimental design. After pre-patching ocular dominance plasticity measurement (baseline), participants underwent 2.5 h of monocular patching with a translucent patch for their dominant eyes. During the patching stage, participants were asked to undertake a gaming task, i.e., playing action video games, watching action video game movies, or playing non-action video games (three tasks on different days). We measured their ocular dominance again at 0, 3, 6, 9, and 30 min (T_1_, T_2_, T_3_, T_4_, T_5_) after the removal of the patch. Note that the sound was turned off when participants were watching action video game movies.

Each participant completed three experiment sessions. In a typical session (Fig. 1), initial ocular dominance was obtained at baseline (T_0_), after which the participant received 2.5-h patching of their dominant eye with a translucent patch. During this stage, one of the three tasks (i.e., playing action video games, watching action video game movies and playing non-action video games), was assigned to each participant. Participants completed the tasks at a comfortable distance depending on the platform of the games (whether desktop or mobile) under normal indoor illuminance. Participants were allowed to take a restroom break as needed; during most of the time, however, participants were instructed to focus on the assigned tasks, under supervision of the experimenter. Subsequently, ocular dominance was measured at 0, 3, 6 and 9 min (T_1_, T_2_, T_3_, T_4_) and 30 min (T_5_) after the patch was removed. These post-patching results were then compared with the initial baseline, and any changes in ocular dominance would indicate the strength of ocular dominance plasticity.

These three experiment sessions (i.e., playing action video games, watching action video game movies and playing non-action video games) were conducted on separate days in a random order. To compare the impact of pure visual stimulation versus that of complex integrated stimulation (e.g., visual stimulation with auditory inputs and attentional engaging), we turned off the sound while participants were watching action video game movies; in this way, action video game movies should be interpreted as movies that provided the same visual inputs as when participants were playing the games. The former condition would enable us to quantify the pure visual plasticity, while the latter would enable us to quantify the additional benefits of playing action video games.

### Procedures

**Figure 2.**
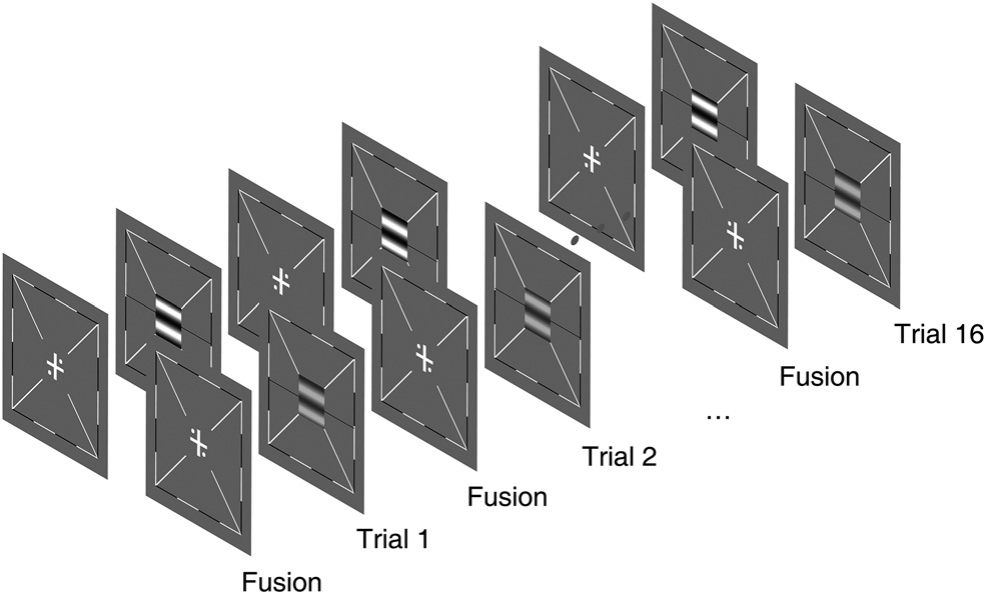
An illustration of the binocular phase combination paradigm. In each trial, participants were first asked to finish an eye alignment (fusion) task and then a binocular phase combination task, where two horizontal gratings with the same spatial frequency (0.46 c/d) and opposite phase shifts from the center of the screen (−22.5° and +22.5°) were dichoptically presented to their two eyes. Participants perceived the two stimuli as one fused horizontal grating, the perceived phase of which was determined by the relative strength of the two eyes’ contributions to the binocular viewing. Stimulus contrast was set as 100% for the non-dominant eye and δ × 100% (0 ≤ δ ≤ 1) for the other eye. δ is the interocular contrast ratio close to individuals’ balance point (i.e., at which the binocular perceived phase was close to zero degrees), which was selected based on their performance from practice trials. Participants were asked to move a flanking reference line to the middle of the central dark stripe of the fused grating. The position of the reference line was then converted into the perceived phase for each participant. To avoid a potential positional bias, two configurations were given: in configuration 1, the phase was set as −22.5° for the dominant eye and +22.5° for the non-dominant eye; in configuration 2, the phase was set reversely. This was repeated eight times in a typical test session, which would last ∼3 min. After all the sixteen trials were performed, an average perceived phase was calculated.

Measurement of ocular dominance was completed by a binocular phase combination paradigm (Ding and Sperling, 2006). In each trial, participants were first asked to finish an eye alignment (fusion) task and then a binocular phase combination task, where two horizontal gratings with the same spatial frequency (0.46 cycles per degree, c/d) and opposite phase shifts (−22.5° and +22.5°) were dichoptically presented to the two eyes of our participants (Fig. 2). Participants would perceive the two stimuli as one fused horizontal grating, of which the perceived phase was determined by the relative strength of the two eyes’ contributions to the binocular viewing. Stimulus contrast was set as 100% for the non-dominant eye and δ × 100% (0 ≤ δ ≤ 1) for the other eye. δ is the interocular contrast ratio close to individuals’ balance point (i.e., at which the binocular perceived phase was close to zero degrees), which was selected based on their performance from practice trials. Participants were asked to move a flanking reference line to the middle of the central dark stripe of the fused grating. This position of the reference line was then converted into the perceived phase for each participant. To avoid a potential positional bias, two configurations were given: in configuration 1, the phase was set as −22.5° for the dominant eye and +22.5° for the non-dominant eye; in configuration 2, the phase was set reversely. This was repeated eight times in a typical test session, which would last ∼3 min. After all the sixteen trials (i.e., two configurations × eight repetitions) were performed, an average perceived phase (i.e., [phase in configuration 1 – phase in configuration 2]/2) was calculated to indicate ocular dominance. A negative change after monocular patching in the perceived phase would indicate that the dominant eye (i.e., the patched eye) became stronger, while a positive change in the perceived phase would indicate that the unpatched eye became stronger. More details of the paradigm (Fig. 2) are described in a previous study (Zhou et al., 2013b).

### Data analysis

We grouped the task of playing action video games and that of watching action video game playing into Comparison 1, and the task of playing action video games and that of playing non-action video games into Comparison 2. The ocular dominance changes at different time sessions after monocular patching were compared by Kruskal–Wallis *H* tests. The results of different tasks in Comparison 1 and Comparison 2, respectively, were compared by repeated-measures ANOVA. To further investigate the magnitude of the effect over time, we calculated the areal measures [area under curve (AUC)] from 0 min to 9 min (i.e., T_1_ to T_4_ in Fig. 1) and performed paired samples *t* tests for further analysis. The level of significance was set as *p *<* *0.05. All statistical analysis was completed in SPSS 23.0 (IBM Corporation).

## Results

### Comparison 1: playing action video games versus watching action video game movies

**Figure 3.**
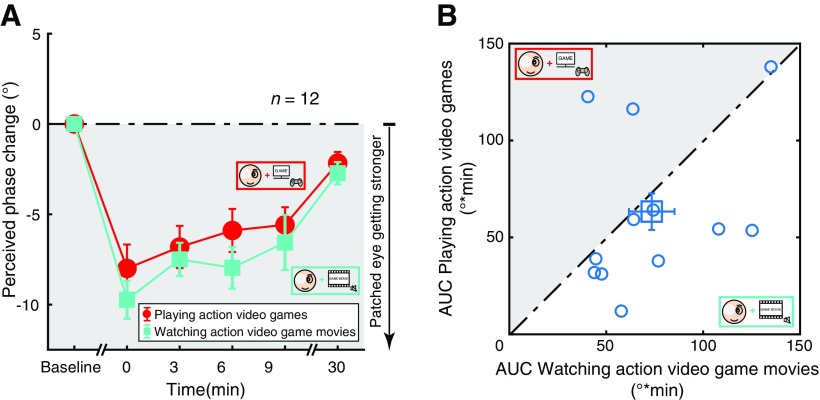
Playing action video games versus watching action video game movies. ***A***, The shift in ocular dominance (i.e., perceived phase change) after monocular patching. Circles represent results of playing action video games during the monocular patching stage; squares represent the results of watching action video game movies during the monocular patching stage. Error bars represent SEs across participants. The dark area suggests a shift of ocular dominance in favor of the patched eye. ***B***, Areal measures (AUC) within the first 10 min (i.e., T_1_ to T_4_ in Fig. 1). The dark area represents a stronger accumulative effect of playing action video games. The blue square represents the average results. Error bars represent SEs across participants.

In Comparison 1, we compare the change in ocular dominance of action video game play during monocular patching with that of action video game movie watching. As shown in Figure 3*A*, participants’ change of ocular dominance (i.e., perceived phase change from the pre-patching baseline) was negative after monocular patching, indicating that the patched eye became stronger in both conditions. Kruskal–Wallis *H* tests also showed that the perceived phase changes were significantly different between different time sessions for both playing action video games (*H*_(5)_ = 39.498, *p *<* *0.001) and watching action video game movies (*H*_(5)_ = 42.798, *p *<* *0.001) conditions. A repeated-measures ANOVA further showed that the perceived phase was not significantly different between the two viewing conditions (*F*_(1,11)_ = 1.122, *p *=**0.312). To better show the difference between the two viewing conditions in different individuals, we also calculated the areal measures (AUC) within the first 10 min (i.e., T_1_ to T_4_ in Fig. 1) and plotted the results in Figure 3*B*. The average effect (i.e., AUC) for the two conditions were 63.34 667 ± 38.71 312 (playing action video games, mean ± SD) and 73.47083 ± 31.04102 (watching action video game movies, mean ± SD). Overall there was no significant difference between the two viewing conditions (*t*_(11)_ = −0.813, *p *=* *0.433; two-tailed paired samples *t* test).

Through a power analysis, we found that the effect size of Comparison 1 was 0.288542, and the sample size needed for power = 80% and significance level = 0.05 would be at least 97. Therefore, we conclude that the difference between the two tasks, if any, would be very small.

### Comparison 2: playing action video games versus playing non-action video games

**Figure 4.**
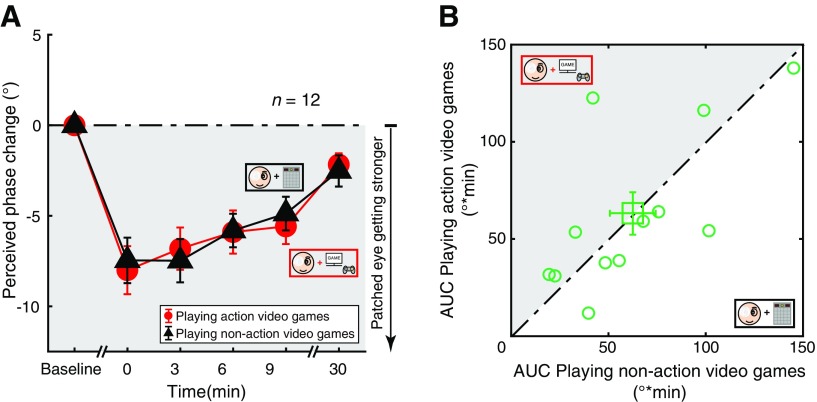
Playing action video games versus playing non-action video games. ***A***, The shift in ocular dominance (i.e., perceived phase change) after monocular patching. Circles represent results of playing action video games during the monocular patching stage; triangles represent results of playing non-action video games during the monocular patching stage. Error bars represent SEs across participants. The dark area suggests a shift of ocular dominance in favor of the patched eye. ***B***, Areal measures (AUC) within the first 10 min (i.e., T_1_ to T_4_ in Fig. 1). The dark area represents a stronger accumulative effect of playing action video games. The green square represents the average results. Error bars represent SEs across participants.

In Comparison 2, we compare the change in ocular dominance of participants playing action video games during monocular patching with that of playing non-action video games. As shown in Figure 4*A*, participants’ perceived phase change from baseline was negative after monocular patching, indicating that the patched eye became stronger in both conditions. Kruskal–Wallis *H* tests also showed that the perceived phase changes were significantly different between different time sessions for both playing action video games (*H*_(5)_ = 39.498, *p *<* *0.001) and playing non-action video games (*H*_(5)_ = 37.250, *p *<* *0.001) conditions. ANOVA further showed that the perceived phase was not significantly different between the two viewing conditions (*F*_(1,11)_ = 0.004, *p *=* *0.951). To better show the difference between the two viewing conditions in different individuals, we calculated the areal measures (AUC) within the first 10 min (i.e., T_1_ to T_4_ in Fig. 1) and plotted the results in Figure 4*B*. The average effect (i.e., AUC) for the two conditions were 63.34667 ± 38.71312 (playing action video games, mean ± SD) and 62.50167 ± 35.82488 (playing non-action video game movies, mean ± SD). There was no significant difference between the two viewing conditions (*t*_(11)_ = 0.092, *p *=* *0.928; two-tailed paired samples *t* test).

Through a power analysis, we found that the effect size of Comparison 2 was 0.022656, and the sample size needed for power = 80% and significance level = 0.05 would be at least 15,294. Therefore, we conclude that there was no difference between these two tasks.

### Does gender play a role in the results?

**Figure 5.**
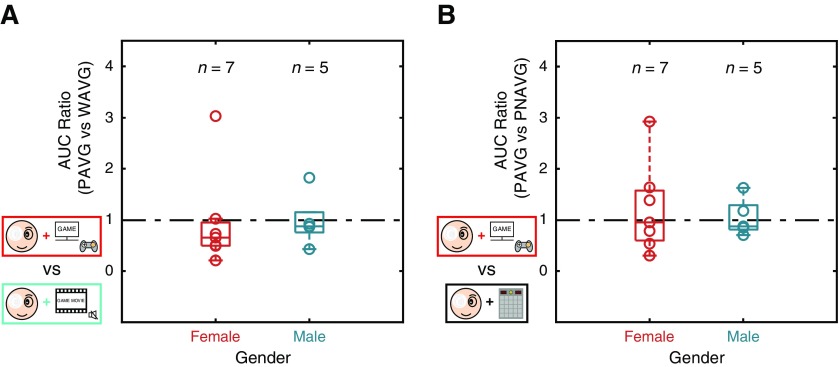
AUC ratio within the first 10 min shown in subgroups of genders in Comparison 1 and Comparison 2. Each circle represents the AUC ratio obtained from one participant. Boxes indicate the medians and the 25th and 75th percentiles of AUC ratio. The factor of gender had no significant role in either Comparison 1 (*t*_(10)_ = −0.074, *p *=* *0.942; ***A***) or Comparison 2 (*t*_(10)_ = 0.405, *p *=* *0.694; ***B***).

One interesting finding in the literature is that male participants might have better performance than females in spatial cognition, while females instead showed larger improvements on the same tasks after action video game training (Feng et al., 2007). To clarify the concern, we classified our participants into two subgroups according to their gender (i.e., male vs female; Fig. 5), and analyzed the AUC ratio between the two tasks in both Comparison 1 and Comparison 2. We found no significant difference between the two subgroups [Comparison 1: *t*_(10)_ = −0.074, *p *=* *0.942 (Fig. 5*A*); Comparison 2: *t*_(10)_ = 0.405, *p *=* *0.694 (Fig. 5*B*)]. These results suggest that the factor of gender had no role in our experiments.

## Discussion

In this study, we investigated whether action video gaming during monocular patching could influence ocular dominance plasticity in visually normal adults. In Comparison 1, we assessed whether there would be a difference in short-term monocular patching induced visual plasticity between two conditions: during monocular patching subjects were either playing (i.e., active attendance) or watching (i.e., passive attendance) action video games. Since the visual stimuli in these two conditions were the same, any difference in short-term monocular patching induced visual plasticity would be due to the outcome of playing action video games. In Comparison 2, we investigated whether there would be a difference in short-term monocular patching induced visual plasticity between when observers were playing either action or non-action video games. A typical action video game is more perceptually demanding and difficult to perform than a non-action video game. Because of this difference, we had hypothesized that action video gaming would exert a larger influence on visual plasticity. However, we found that patching with playing action video games did not enhance or eliminate the magnitude of ocular dominance change in either Comparison 1 or Comparison 2. Eye fatigue was not monitored in this experiment; however, some participants did report eye fatigue after playing action video games while others did not.

As a novel form of visual plasticity, short-term ocular dominance plasticity, with its effect on binocular balance and potential for amblyopic treatment (Zhou et al., 2013b, 2019; Lunghi et al., 2019), has drawn the attention of many scientists in the field of vision science. However, the change is transient, as opposed to the permanence of the neuroplastic changes that occur from long-term monocular deprivation during the critical period (Daw, 2014). Recent investigators have postulated that that these two forms of neural plasticity in the visual system are related. For instance, Lunghi et al. (2016) argued that the plasticity induced by short-term monocular patching could predict that induced by long-term patching, thus suggesting a similar neural mechanism for the two types of plasticity. Sauvan et al. (2019) reported that short-term monocular patching could enhance the effect of long-term plasticity, albeit not significantly. In addition, action video gaming has been reported to improve perceptual performance on visual tasks after a few weeks or months of visual training (Levi and Li, 2009; Li et al., 2013; Vedamurthy et al., 2015; Gambacorta et al., 2018). This form of improvement is called perceptual learning. If action video gaming could enhance changes in visual plasticity, it could be employed in concert with monocular patching and monocular training to improve the visual acuity as well as binocular balance in patients with poor vision and other visual disorders such as amblyopia (Gambacorta et al., 2018).

The finding is interesting that there is no significant difference between action video game play and non-action video game play in our participants in terms of short-term ocular dominance plasticity. It is worth noting that in a previous study, Li and colleagues demonstrated a larger improvement in contrast sensitivity with action video game training than with non-action video game training in visually normal adults (Li et al., 2009). It is likely that such an improvement reflects a change in monocular sensitivity in the early cortical pathway (e.g., V1) and is relevant to perceptual learning. Therefore, the inconsistency between our study and Li and colleagues’ might be due to different neural mechanisms being responsible for perceptual learning and monocular patching-induced plasticity. Perceptual learning relies on repeated intensively visual training and is thought to involve the properties (i.e., peak tuning and signal/noise) of individual cortical neurons before binocular summation (Hua et al., 2010; Ren et al., 2016), whereas monocular patching-induced plasticity relies on short-term visual deprivation and involves the interactions between neurons receiving left and right eye inputs (Binda et al., 2018; Chadnova et al., 2017; Zhou et al., 2015).

We had expected to see an enhancement of ocular dominance change by action video game play for two reasons. First, during patching, playing action video games via the unpatched eye could recruit additional attentive processes involving top-down feedback from higher visual areas (Gilbert and Li, 2013). If such attentional feedback modulated changes in ocular dominance, a low-level phenomenon, the change in ocular dominance would have increased. However, this was not the case in our findings from Comparison 2. Hence, ocular dominance plasticity seems to be determined by local low-level, feedforward interactions in the primary visual cortex. Second, cross-modal inputs have been shown to affect visual plasticity (Iurilli et al., 2012; Ibrahim et al., 2016; Teichert et al., 2018, 2019) by suppressing the early visual cortical activity in animals (Iurilli et al., 2012; Ibrahim et al., 2016). Also, recent studies have revealed that non-visual sensory deprivation can cross-modally restore plasticity in the visual cortex in matured mice (Teichert et al., 2018, 2019). Nevertheless, our results in the two comparisons show that complex integrated stimulation (e.g., auditory inputs and attentional engaging) seems to exert no additional effect, compared with visual stimulation alone (i.e., watching without hearing or playing), on short-term ocular dominance plasticity in human adults. Therefore, the neural mechanisms responsible for cross-modal influences may not be involved in the neural plasticity induced by short-term monocular patching. Another explanation, however, is that the attentional engagement and the visuo-auditory integration might have opposite effects on short-term ocular dominance plasticity, which could lead to a null effect as observed in our experiment. Future studies may need to determine the separate effects of these factors.

In fact, our finding that the complex integrated stimulation may not play a significant role in short-term ocular dominance plasticity is consistent with the ones from other studies which demonstrate that only low-level areas are locally involved in short-term ocular dominance plasticity in human adults (Lunghi et al., 2015a,b; Chadnova et al., 2017; Binda et al., 2018). To illustrate, an electrophysiological study reports a change in the amplitude of the C1 component following patching, a phenomenon that has been confirmed to be closely related to the activity of V1 (Lunghi et al., 2015a). There is also evidence that reduced GABA concentration in V1 is highly correlated with the perceptual boost of the patched eye (Lunghi et al., 2015b). Furthermore, a recent fMRI study suggests a significant impact on the neural coding at the level of V1 after 2 h of monocular contrast deprivation (Binda et al., 2018). Our results, together with these previous reports, suggest that the neural site of the ocular dominance plasticity from monocular patching is local and resides within the early cortical pathway.

In short, we found that action video gaming does not impact short-term ocular dominance plasticity in visually normal adults more than watching action video game movies or playing non-action video games. Thus, complex integrated stimulation, in contrast to visual stimulation alone, may not play a significant role in this plasticity in human adults. Our findings suggest the neural mechanism responsible for short-term ocular dominance plasticity might occur early in the cortical pathway.

## Synthesis

Reviewing Editor: Christine Portfors, Washington State University Decisions are customarily a result of the Reviewing Editor and the peer reviewers coming together and discussing their recommendations until a consensus is reached. When revisions are invited, a fact-based synthesis statement explaining their decision and outlining what is needed to prepare a revision will be listed below. The following reviewer(s) agreed to reveal their identity: Gianluca Campana.

Synthesis:

While the reviewers think the manuscript is interesting, a lack of detail in the procedures makes interpretation of the data challenging. The validity of the claims are difficult to judge which dramatically reduces the impact of the study. The manuscript could be improved substantially with a much more detailed description of the procedures and a more appropriately structured discussion.

Reviewer’s Comments:

Reviewer 1

This manuscript assesses whether action video gaming, known to enhance perceptual learning and boosts plasticity, is also able to modulate a low-level and short-term type of plasticity (namely: a shift in ocular dominance). Results show that there is no interaction between action videogaming and this low-level, short-term type of plasticity. This suggests that shifts of ocular dominance occur early in the cortical pathway and operate at a local level.

The topic is interesting, the paper well written.

I only have a couple of points.

Page 4: Please specify what Ethics Committee approves this project.

Page 13: The very last sentence starting with “Our psychophysical findings” is not clear and should be rephrased.

More in general: it is not completely clear why one would expect that a procedure that boosts long-term perceptual learning and neural plasticity should have any effect on a short-term, reversible type of plasticity.

Reviewer 2

My main concern is that the procedures of this study are very poorly described. In particular, nothing is said about the playing/watching conditions during the patching whereas these are crucial to interpret the results and draw general conclusions. Worse, this lack of description suggests the study is poorly controlled (was the engagement evaluated ? why removing the sound in the watching condition ?).

Therefore, it is difficult to judge the validity of the claims. These caveats prevents from drawing more general conclusions on plasticity mechanisms and how attention/engagement or multisensory integration can affect this effect which is really unsatisfactory whereas it could have been much more impactful.

intro

last paragraph of the intro: the rationale is not well explained. Why testing videogames is interesting ? what is specific about action video games ? How attention and plasticity mechanisms would be combined ? What are the hypothesis and expectations ?

methods

The procedures and methods are insufficiently described. We basically don’t know what happened during the patching time and how the activity of the participants was monitored/controlled.

How did you evaluate participants’ engagement in the games ? Were they really focused on the game ? Were they supervised ?

No details at all are provided about the playing/watching sessions !

Was it on a computer or a tablet ? Type of screen ? Which field of view ? Distance/ luminosity of screen. What was the illuminance of the room ? Were people alone in the room ? How many breaks did they take ? Could they be distracted ? Did they report eye fatigue ? Etc ?

No details are given about participants ? Were they experienced players ? It might help to estimate their engagement

line 140: “It should be noted that we turned off the sound while the action video game movies were being watched.”

Sound was on during game playing. Why turning off the sound only in this condition ?

Were people in a quiet room then, or there were disturbing noises ?

Line 142: “Subjects were only provided with visual input - but no auditory input - that matched the playing of action video games. “

I don’t understand this sentence

line 254: “In this study, we designed three different tasks and grouped them into two experiments"

This is misleading. You say there were 2 experiments, but the data from the PAVG condition seems to be the same in the 2 experiments. So it is only one experiment ! But just 2 Comparisons. Also you don’t say in which order conditions were tested.

In the “experiment 1” it looks there could be a difference between the 2 conditions. Did you run a power analysis in order to know how many subjects you would need to show an effect ?

Discussion

The discussion is interesting but not appropriately structured. Also it should be extended regarding how the mechanisms between patching and perceptual learning would combine in regard of the hypothesis

line 293: “Our results in the two experiments, however, show that cross-modal stimulation (e.g. sound of the game and the engagement of playing) does not influence visual input alone (i.e. watching without hearing or playing)"

You can’t discriminate because, as far as I understand, you removed both audio and playing inputs at the same time in the watching condition

The differences between the 3 tasks are not well controlled (or at leat not well explained). So we don’t know about engagement differences between the different tasks. Thus it is not possible to deduce a general conclusion about involvement of top-down effects.

Line 317: “Attentional top-down influences as well as cross-modal processes do not seem to influence this form of ocular dominance plasticity in human adults.”

You can’t conclude that, because as it is, we can’t know if your participants were really paying attention while playing.

Minor

Line 7: “.5 to 5 hours” whereas you say line 23: “.5 to 3 hours"

line 73 and several others places: Lot of relevant references from various groups are missing, but I guess it is on purpose to try to keep anonymity

Fig 5: this is a ratio, so the reference line should be put at 1 ! Or even better, the ratio should be expressed in dB. this way the figure is very misleading.

## References

[B1] Appelbaum LG, Cain MS, Darling EF, Mitroff SR (2013) Action video game playing is associated with improved visual sensitivity, but not alterations in visual sensory memory. Atten Percept Psychophys 75:1161–1167. 10.3758/s13414-013-0472-7 23709062

[B2] Bai J, Dong X, He S, Bao M (2017) Monocular deprivation of Fourier phase information boosts the deprived eye’s dominance during interocular competition but not interocular phase combination. Neuroscience 352:122–130. 10.1016/j.neuroscience.2017.03.053 28391010

[B3] Başgöze Z, Mackey AP, Cooper EA (2018) Plasticity and adaptation in adult binocular vision. Curr Biol 28:R1406–R1413. 10.1016/j.cub.2018.10.024 30562537

[B4] Bavelier D, Green CS (2019) Enhancing attentional control: lessons from action video games. Neuron 104:147–163. 10.1016/j.neuron.2019.09.031 31600511

[B5] Bediou B, Adams DM, Mayer RE, Tipton E, Green CS, Bavelier D (2018) Meta-analysis of action video game impact on perceptual, attentional, and cognitive skills. Psychol Bull 144:77–110. 10.1037/bul0000130 29172564

[B6] Berardi N, Pizzorusso T, Maffei L (2000) Critical periods during sensory development. Curr Opin Neurobiol 10:138–145. 10.1016/s0959-4388(99)00047-1 10679428

[B7] Binda P, Kurzawski JW, Lunghi C, Biagi L, Tosetti M, Morrone MC (2018) Response to short-term deprivation of the human adult visual cortex measured with 7T BOLD. Elife 7 10.7554/eLife.40014 PMC6298775 30475210

[B8] Bisoglio J, Michaels TI, Mervis JE, Ashinoff BK (2014) Cognitive enhancement through action video game training: great expectations require greater evidence. Front Psychol 5:136. 24600427 10.3389/fpsyg.2014.00136 PMC3928536

[B9] Blacker KJ, Curby KM (2013) Enhanced visual short-term memory in action video game players. Atten Percept Psychophys 75:1128–1136. 10.3758/s13414-013-0487-0 23709068

[B10] Boot WR, Kramer AF, Simons DJ, Fabiani M, Gratton G (2008) The effects of video game playing on attention, memory, and executive control. Acta Psychol (Amst) 129:387–398. 10.1016/j.actpsy.2008.09.005 18929349

[B11] Buckley D, Codina C, Bhardwaj P, Pascalis O (2010) Action video game players and deaf observers have larger Goldmann visual fields. Vision Res 50:548–556. 10.1016/j.visres.2009.11.018 19962395

[B12] Castel AD, Pratt J, Drummond E (2005) The effects of action video game experience on the time course of inhibition of return and the efficiency of visual search. Acta Psychol (Amst) 119:217–230. 10.1016/j.actpsy.2005.02.004 15877981

[B13] Chadnova E, Reynaud A, Clavagnier S, Hess RF (2017) Short-term monocular occlusion produces changes in ocular dominance by a reciprocal modulation of interocular inhibition. Sci Rep 7:41747. 10.1038/srep41747 28150723 PMC5288724

[B14] Dale G, Green CS (2017) The changing face of video games and video gamers: future directions in the scientific study of video game play and cognitive performance. J Cogn Enhanc 1:280–294. 10.1007/s41465-017-0015-6

[B15] Daw NW (2014) Visual development, Ed 3 New York: Springer.

[B16] Ding J, Sperling G (2006) A gain-control theory of binocular combination. Proc Natl Acad Sci USA 103:1141–1146. 10.1073/pnas.0509629103 16410354 PMC1347993

[B17] Dye MWG, Green CS, Bavelier D (2009) The development of attention skills in action video game players. Neuropsychologia 47:1780–1789. 10.1016/j.neuropsychologia.2009.02.002 19428410 PMC2680769

[B18] Feng J, Spence I, Pratt J (2007) Playing an action video game reduces gender differences in spatial cognition. Psychol Sci 18:850–855. 10.1111/j.1467-9280.2007.01990.x 17894600

[B19] Föcker J, Cole D, Beer AL, Bavelier D (2018) Neural bases of enhanced attentional control: lessons from action video game players. Brain Behav 8:e01019. 10.1002/brb3.1019 29920981 PMC6043695

[B20] Franceschini S, Trevisan P, Ronconi L, Bertoni S, Colmar S, Double K, Facoetti A, Gori S (2017) Action video games improve reading abilities and visual-to-auditory attentional shifting in English-speaking children with dyslexia. Sci Rep 7:5863. 10.1038/s41598-017-05826-8 28725022 PMC5517521

[B21] Gambacorta C, Nahum M, Vedamurthy I, Bayliss J, Jordan J, Bavelier D, Levi DM (2018) An action video game for the treatment of amblyopia in children: a feasibility study. Vision Res 148:1–14. 10.1016/j.visres.2018.04.005 29709618 PMC5984723

[B22] Gilbert CD, Li W (2013) Top-down influences on visual processing. Nat Rev Neurosci 14:350–363. 10.1038/nrn3476 23595013 PMC3864796

[B23] Green CS, Bavelier D (2003) Action video game modifies visual selective attention. Nature 423:534–537. 10.1038/nature01647 12774121

[B24] Green CS, Bavelier D (2006) Enumeration versus multiple object tracking: the case of action video game players. Cognition 101:217–245. 10.1016/j.cognition.2005.10.004 16359652 PMC2896820

[B25] Green CS, Bavelier D (2007) Action-video-game experience alters the spatial resolution of vision. Psychol Sci 18:88–94. 10.1111/j.1467-9280.2007.01853.x 17362383 PMC2896830

[B26] Green CS, Bavelier D (2012) Learning, attentional control, and action video games. Curr Biol 22:R197–R206. 22440805 10.1016/j.cub.2012.02.012 PMC3461277

[B27] Hua T, Bao P, Huang C-B, Wang Z, Xu J, Zhou Y, Lu Z-L (2010) Perceptual learning improves contrast sensitivity of V1 neurons in cats. Curr Biol 20:887–894. 10.1016/j.cub.2010.03.066 20451388 PMC2877770

[B28] Huang V, Young M, Fiocco AJ (2017) The association between video game play and cognitive function: does gaming platform matter? Cyberpsychol Behav Soc Netw 20:689–694. 10.1089/cyber.2017.0241 29048933

[B29] Hubel DH, Wiesel TN (1970) The period of susceptibility to the physiological effects of unilateral eye closure in kittens. J Physiol 206:419–436. 10.1113/jphysiol.1970.sp009022 5498493 PMC1348655

[B30] Ibrahim LA, Mesik L, Ji X, Fang Q, Li H, Li Y, Zingg B, Zhang LI, Tao HW (2016) Cross-modality sharpening of visual cortical processing through layer-1-mediated inhibition and disinhibition. Neuron 89:1031–1045. 10.1016/j.neuron.2016.01.027 26898778 PMC4874809

[B31] Iurilli G, Ghezzi D, Olcese U, Lassi G, Nazzaro C, Tonini R, Tucci V, Benfenati F, Medini P (2012) Sound-driven synaptic inhibition in primary visual cortex. Neuron 73:814–828. 10.1016/j.neuron.2011.12.026 22365553 PMC3315003

[B32] Jeon ST, Maurer D, Lewis TL (2012) The effect of video game training on the vision of adults with bilateral deprivation amblyopia. Seeing Perceiving 25:493–520. 10.1163/18784763-00002391 23193607

[B33] Kim HW, Kim CY, Blake R (2017) Monocular perceptual deprivation from interocular suppression temporarily imbalances ocular dominance. Curr Biol 27:884–889. 10.1016/j.cub.2017.01.063 28262490

[B34] Latham AJ, Patston LLM, Westermann C, Kirk IJ, Tippett LJ (2013) Earlier visual N1 latencies in expert video-game players: a temporal basis of enhanced visuospatial performance? PLoS One 8:e75231. 10.1371/journal.pone.0075231 24058667 PMC3776734

[B35] Levi DM, Li RW (2009) Perceptual learning as a potential treatment for amblyopia: a mini-review. Vision Res 49:2535–2549. 10.1016/j.visres.2009.02.010 19250947 PMC2764839

[B36] Li J, Thompson B, Deng D, Chan LYL, Yu M, Hess RF (2013) Dichoptic training enables the adult amblyopic brain to learn. Curr Biol 23:R308–R309. 10.1016/j.cub.2013.01.059 23618662

[B37] Li R, Polat U, Makous W, Bavelier D (2009) Enhancing the contrast sensitivity function through action video game training. Nat Neurosci 12:549–551. 10.1038/nn.2296 19330003 PMC2921999

[B38] Li RW, Ngo C, Nguyen J, Levi DM (2011) Video-game play induces plasticity in the visual system of adults with amblyopia. PLoS Biol 9:e1001135. 10.1371/journal.pbio.1001135 21912514 PMC3166159

[B39] Lunghi C, Burr DC, Morrone MC (2011) Brief periods of monocular deprivation disrupt ocular balance in human adult visual cortex. Curr Biol 21:R538–R539. 10.1016/j.cub.2011.06.004 21783029

[B40] Lunghi C, Berchicci M, Morrone MC, Di Russo F (2015a) Short-term monocular deprivation alters early components of visual evoked potentials. J Physiol 593:4361–4372. 10.1113/JP270950 26119530 PMC4594246

[B41] Lunghi C, Emir UE, Morrone MC, Bridge H (2015b) Short-term monocular deprivation alters GABA in the adult human visual cortex. Curr Biol 25:1496–1501. 10.1016/j.cub.2015.04.021 26004760 PMC5040500

[B42] Lunghi C, Morrone MC, Secci J, Caputo R (2016) Binocular rivalry measured 2 hours after occlusion therapy predicts the recovery rate of the amblyopic eye in anisometropic children. Invest Ophthalmol Vis Sci 57:1537–1546. 10.1167/iovs.15-18419 27046118 PMC4909145

[B43] Lunghi C, Sframeli AT, Lepri A, Lepri M, Lisi D, Sale A, Morrone MC (2019) A new counterintuitive training for adult amblyopia. Ann Clin Transl Neurol 6:274–284. 10.1002/acn3.698 30847360 PMC6389748

[B44] Min SH, Baldwin AS, Reynaud A, Hess RF (2018) The shift in ocular dominance from short-term monocular deprivation exhibits no dependence on duration of deprivation. Sci Rep 8:17083. 10.1038/s41598-018-35084-1 30459412 PMC6244356

[B45] Min SH, Baldwin AS, Hess RF (2019) Ocular dominance plasticity: a binocular combination task finds no cumulative effect with repeated patching. Vision Res 161:36–42. 10.1016/j.visres.2019.05.007 31194984

[B46] Morin-Moncet O, Therrien-Blanchet J-M, Ferland MC, Théoret H, West GL (2016) Action video game playing is reflected in enhanced visuomotor performance and increased corticospinal excitability. PLoS One 11:e0169013. 10.1371/journal.pone.0169013 28005989 PMC5179116

[B47] Oei AC, Patterson MD (2013) Enhancing cognition with video games: a multiple game training study. PLoS One 8:e58546 10.1371/journal.pone.0058546 23516504 PMC3596277

[B48] Ramamurthy M, Blaser E (2018) Assessing the kaleidoscope of monocular deprivation effects. J Vis 18:14. 10.1167/18.13.14 30572342

[B49] Ren Z, Zhou J, Yao Z, Wang Z, Yuan N, Xu G, Wang X, Zhang B, Hess RF, Zhou Y (2016) Neuronal basis of perceptual learning in striate cortex. Sci Rep 6:24769. 10.1038/srep24769 27094565 PMC4837366

[B50] Sauvan L, Stolowy N, Denis D, Matonti F, Chavane F, Hess RF, Reynaud A (2019) Contribution of short-time occlusion of the amblyopic eye to a passive dichoptic video treatment for amblyopia beyond the critical period. Neural Plast 2019:6208414 10.1155/2019/6208414 31558900 PMC6735187

[B51] Teichert M, Isstas M, Zhang Y, Bolz J (2018) Cross-modal restoration of ocular dominance plasticity in adult mice. Eur J Neurosci 47:1375–1384. 10.1111/ejn.13944 29761580

[B52] Teichert M, Isstas M, Liebmann L, Hübner CA, Wieske F, Winter C, Lehmann K, Bolz J (2019) Visual deprivation independent shift of ocular dominance induced by cross-modal plasticity. PLoS One 14:e0213616. 10.1371/journal.pone.0213616 30856226 PMC6411125

[B53] Vedamurthy I, Nahum M, Huang SJ, Zheng F, Bayliss J, Bavelier D, Levi DM (2015) A dichoptic custom-made action video game as a treatment for adult amblyopia. Vision Res 114:173–187. 10.1016/j.visres.2015.04.008 25917239 PMC4549206

[B54] Wang P, Liu HH, Zhu XT, Meng T, Li HJ, Zuo XN (2016) Action video game training for healthy adults: a meta-analytic study. Front Psychol 7:907. 10.3389/fpsyg.2016.00907 27378996 PMC4911405

[B55] Wilms IL, Petersen A, Vangkilde S (2013) Intensive video gaming improves encoding speed to visual short-term memory in young male adults. Acta Psychol (Amst) 142:108–118. 10.1016/j.actpsy.2012.11.003 23261420

[B56] Wong NHL, Chang DHF (2018) Attentional advantages in video-game experts are not related to perceptual tendencies. Sci Rep 8:5528. 10.1038/s41598-018-23819-z 29615743 PMC5882918

[B57] Zhou J, Clavagnier S, Hess RF (2013a) Short-term monocular deprivation strengthens the patched eye’s contribution to binocular combination. J Vis 13:12–12. 10.1167/13.5.12 23599416

[B58] Zhou J, Thompson B, Hess RF (2013b) A new form of rapid binocular plasticity in adult with amblyopia. Sci Rep 3:2638. 10.1038/srep02638 24026421 PMC3770967

[B59] Zhou J, Baker DH, Simard M, Saint-Amour D, Hess RF (2015) Short-term monocular patching boosts the patched eye’s response in visual cortex. Restor Neurol Neurosci 33:381–387. 10.3233/RNN-140472 26410580 PMC4923712

[B60] Zhou J, Liu Z, Clavagnier S, Reynaud A, Hou F (2017) Visual plasticity in adults. Neural Plast 2017:8469580. 10.1155/2017/8469580 28660083 PMC5474249

[B61] Zhou J, He Z, Wu Y, Chen Y, Chen X, Liang Y, Mao Y, Yao Z, Lu F, Qu J, Hess RF (2019) Inverse occlusion: a binocularly motivated treatment for amblyopia. Neural Plast 2019:5157628 10.1155/2019/5157628 31015829 PMC6444262

